# Anomalous middle turbinate with choanal obstruction and maxillary sinusitis: a case report

**DOI:** 10.1186/1752-1947-7-242

**Published:** 2013-10-17

**Authors:** Yong-Wan Kim, Jung-Hoon Lee, Sung-Lyong Hong, Kyu-Sup Cho

**Affiliations:** 1 Department of Otorhinolaryngology, Inje University College of Medicine, Haeundae Paik Hospital, Busan, Republic of Korea; 2 Department of Otorhinolaryngology and Biomedical Research Institute, Pusan National University School of Medicine, 1-10 Ami-dong, Seo-gu, Busan 602-739, Republic of Korea

**Keywords:** Congenital abnormalities, Maxillary sinusitis, Nasal obstruction, Nasopharynx, Turbinates

## Abstract

**Introduction:**

Although several anatomical anomalies in the middle turbinate have been reported, they usually remain asymptomatic. However, complicated aberrant middle turbinate extending through the choana to the contralateral nasopharynx has not been reported.

**Case presentation:**

A 23-year-old Korean woman presented with a 6-month history of nasal obstruction and postnasal discharge refractory to medical treatment. An endoscopic examination revealed an abnormal middle turbinate, and a pedunculated nasal mass covered with normal mucosa that originated from the right lateral nasal wall filled most of the right posterior choana and extended to the left nasopharynx across the posterior free edge of the nasal septum. Computed tomography of the paranasal sinus showed abnormal bony trabeculation from the posterior bony attachment of the right middle turbinate to the left posterior nasopharyngeal wall. In addition, right maxillary sinusitis was observed. From these findings, the lesion was suspected to be an abnormal configuration of the right middle turbinate with secondary maxillary sinusitis and was successfully treated by resection of the malformed middle turbinate and middle meatal antrostomy.

**Conclusions:**

Although this case illustrates a very rare developmental anomaly of the middle turbinate, thorough knowledge of the development of and anatomical anomalies associated with the middle turbinate is of utmost importance to perform proper sinus surgery and avoid complications. Therefore, these findings should be considered by otolaryngologists, and careful examination of the nasal cavity is necessary to determine the existence of this rare anomaly.

## Introduction

The middle turbinate (MT) is an important anatomical structure extending from the lateral nasal wall into the nasal cavity [1]. Although concha bullosa is the most frequently encountered anatomical anomaly in the MT, many others, including paradoxical, secondary, and accessory MT as well as agenesis of the MT, have been reported [1-3]. However, an aberrantly configured MT extending into the nasopharynx occurs less frequently, and a complicated aberrant MT extending through the choana to the contralateral nasopharynx has not been reported to the best of our knowledge. Here, we describe a rare clinical presentation of anomalous MT with choanal obstruction and secondary maxillary sinusitis.

## Case presentation

**Figure 1:**
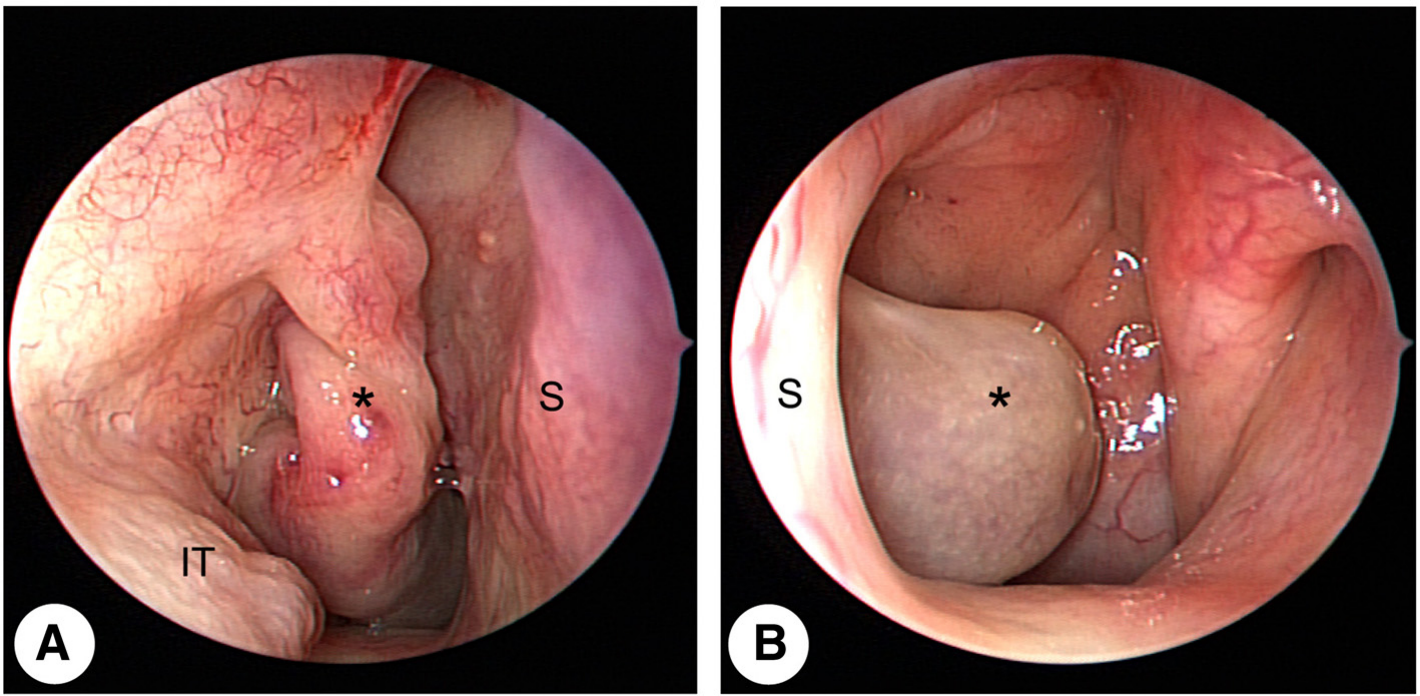
**Preoperative endoscopic findings. (A)** Endoscopic view of the right nasal cavity shows a pedunculated nasal mass (asterisk) covered with normal mucosa attached to the right lateral nasal wall, which filled most of the posterior choana. **(B)** Endoscopic view of the left nasal cavity shows the mass extending to the left nasopharynx across the posterior free edge of the nasal septum. IT: inferior turbinate, S: septum.

**Figure 2:**
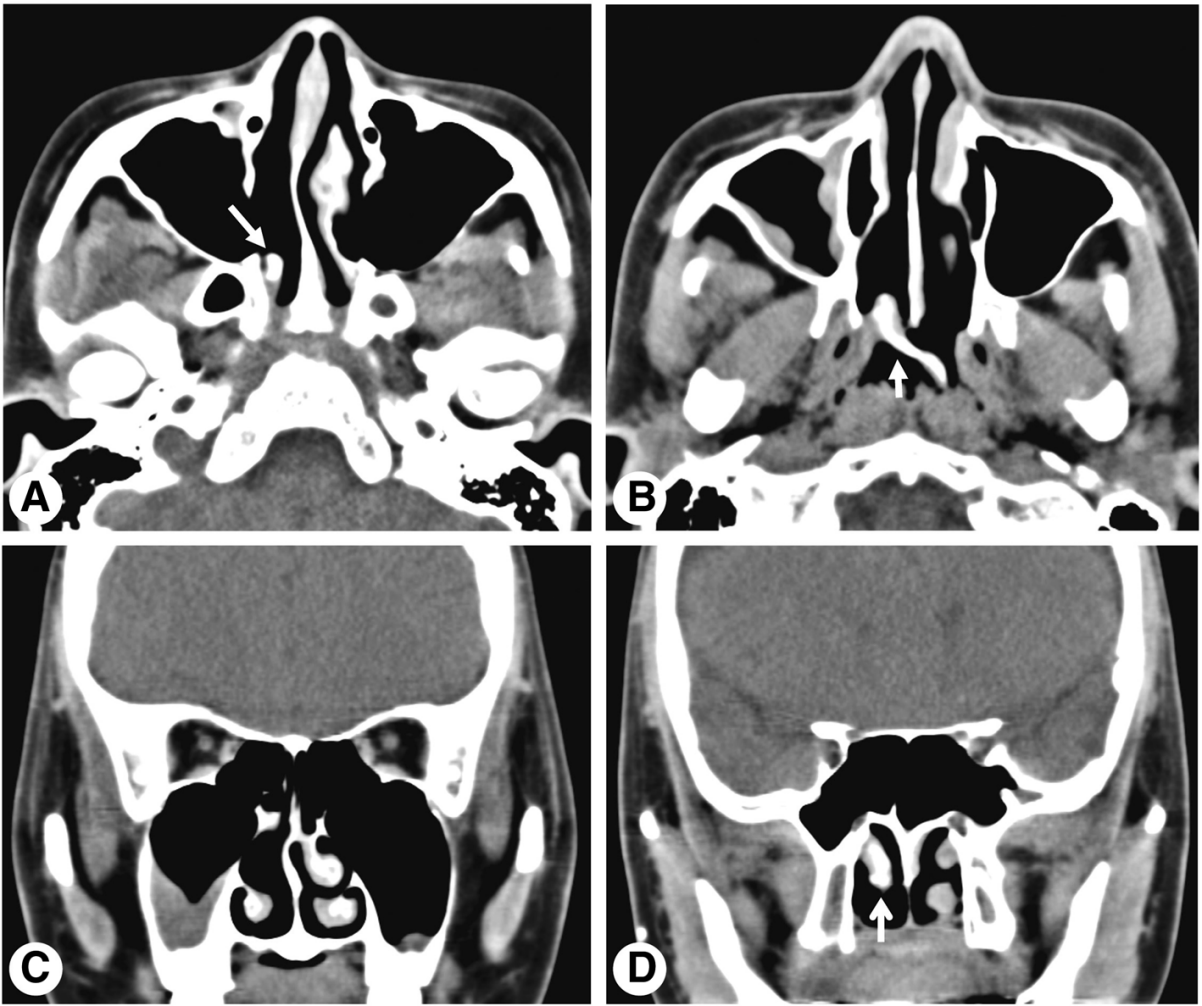
**Preoperative computed tomography images of the paranasal sinus. (A, B)** Axial computed tomography images show abnormal bony trabeculation (arrow) from the posterior bony attachment of the right middle turbinate to the left posterior nasopharyngeal wall. Coronal computed tomography images show anterior agenesis of the right middle turbinate and right maxillary sinusitis **(C)**, and the right middle turbinate (arrow) extending through the right choana **(D)**.

**Figure 3:**
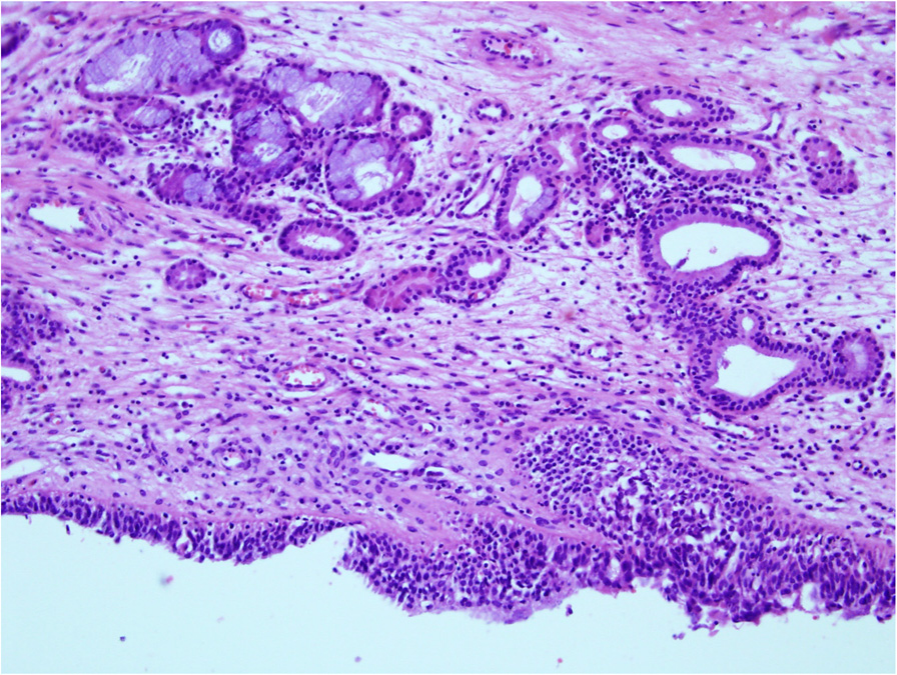
**Histopathological finding of a malformed middle turbinate.** High-power view of the turbinate mucosa shows hyperplastic respiratory epithelium, subepithelial inflammation, and mucosal mucus glands (hematoxylin and eosin, ×200).

**Figure 4:**
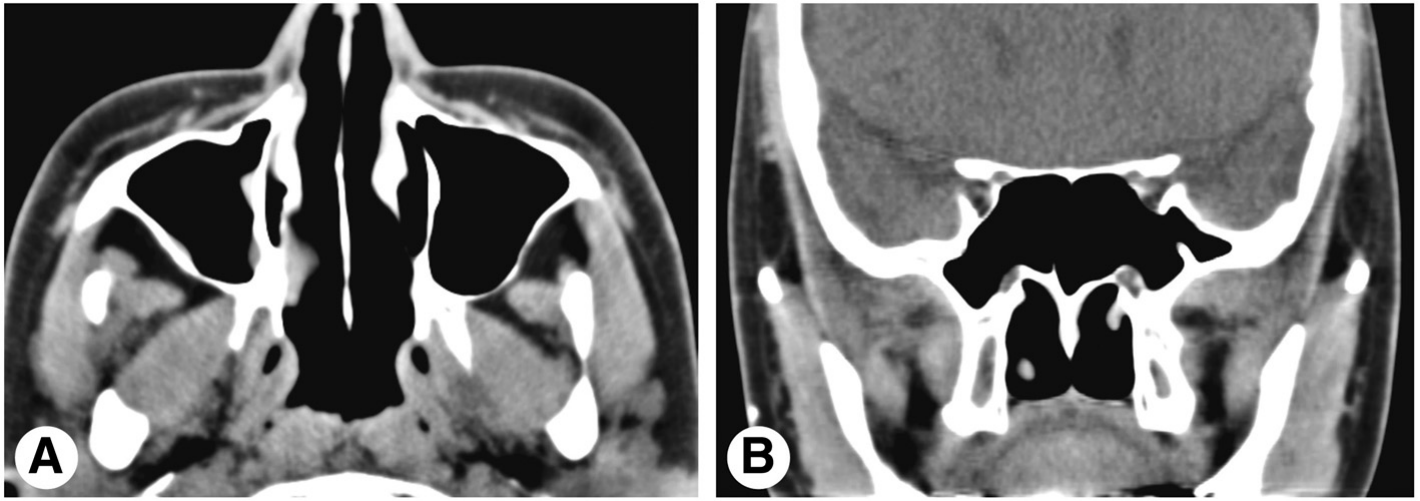
**Postoperative computed tomography images of the paranasal sinus.** Axial **(A)** and coronal **(B)** computed tomography images at 1 month postoperatively show complete resolution of the malformed middle turbinate and right maxillary sinusitis.

A 23-year-old Korean woman presented with a 6-month history of nasal obstruction and postnasal discharge refractory to medical treatment. She had no history of nasal surgery and trauma. An endoscopic examination revealed an abnormal MT, and a pedunculated nasal mass covered with normal mucosa that originated from the right lateral nasal wall filled most of the right posterior choana and extended to the left nasopharynx across the posterior free edge of the nasal septum (Figure 1). Computed tomography (CT) of the paranasal sinus showed abnormal bony trabeculation from the posterior bony attachment of the right MT to the left posterior nasopharyngeal wall. In addition, right maxillary sinusitis was observed (Figure 2). From these findings, the lesion was suspected to be an abnormal configuration of the right MT with secondary maxillary sinusitis. Resection of the malformed MT and middle meatal antrostomy were performed under general anesthesia. The pedunculated MT was transected at the pedicle attached to the lateral nasal wall and delivered out through the right nostril. The natural ostium of the maxillary sinus was patent, and a discharge of pus and inflammation-induced edematous sinus mucosa were observed through the antrostomy site. There were no other malformations in the uncinate process and anterior ethmoid sinus. A histopathological examination of the specimen was conclusive for benign respiratory mucosa with a firm bony structure (Figure 3). Oral antibiotic treatment with amoxicillin and clavulanate was started for concurrent paranasal sinus infection and was planned to be completed in 2 weeks. The patient’s symptoms quickly diminished postoperatively, and follow-up CT 1 month after surgery demonstrated complete resolution of malformed MT and right maxillary sinusitis (Figure 4).

## Discussion

The precursor structures of nasal turbinates appear between the 8th and 10th weeks of fetal life and are the ethmoturbinal and maxilloturbinal [1,4]. The maxilloturbinal develops into the inferior turbinate, whereas the permanent MT and superior turbinate develop from the third and fourth ethmoturbinal, respectively [1,4]. During this developmental process, many anatomical anomalies may arise, but most usually remain asymptomatic. The present case illustrates a congenital anomaly of MT causing choanal obstruction and maxillary sinusitis.

The MT is a complex, three-dimensional structure whose overall shape may not be perceived initially. Its anterior portion inserts into the ascending process of the maxilla and the posteromedial margin of the agger nasi cells. Its superior insertion is to the lateral lamella of the cribriform plate of the ethmoid bone [5]. The posterior bony attachment of the MT to the lateral nasal wall occurs at the crista ethmoidalis of the perpendicular process of the palatine bone, which is often used as an anatomic marker anterior to the sphenopalatine foramen [5]. Our case showed agenesis of the anterior portion of the MT and a malformed MT that obliquely extended from the posterior bony attachment to the contralateral nasopharynx across the posterior free edge of the nasal septum.

The MT plays functional roles in nasal physiology, including humidification of inspired air, lamination of the airflow, and deflection of inspired air superiorly toward the olfactory epithelium [1,3]. In particular, the structure and dimensions of the MT are crucial to the maintenance of normal nasal physiology. Secretions produced by the nasal and paranasal sinus mucosal lining are transported posteriorly along the surfaces of the MT to reach the nasopharynx. Therefore, anatomical anomalies in the MT may disrupt this process, resulting in secondary maxillary sinusitis. In addition to its physiological significance, the MT is often used as a reference point during endoscopic sinus surgery [6]. Therefore, awareness of anatomical anomalies of the MT is very important for otolaryngologists evaluating patients who have nasal and paranasal sinus disease. In our case, nasal obstruction and postnasal discharge were thought to be caused by choanal obstruction of a malformed MT, altering the airflow and obstructing the drainage of the sinonasal secretions.

Although the anatomical anomalies in this patient are very rare and mimicked a nasopharyngeal mass, both endoscopic and CT findings supported the diagnosis of an anomalous MT with choanal obstruction and maxillary sinusitis that was successfully treated by endoscopic excision and middle meatal antrostomy.

## Conclusions

Although this case illustrates a very rare developmental anomaly of the MT, thorough knowledge of the development of and anatomical anomalies associated with the MT is of utmost importance to perform proper sinus surgery and avoid complications. Furthermore, this anatomical anomaly can lead to significant choanal obstruction with resultant maxillary sinusitis. Therefore, these findings should be considered by otolaryngologists, and careful examination of the nasal cavity should be carried out to determine the existence of this rare anomaly.

## Consent

Written informed consent was obtained from the patient for publication of this manuscript and accompanying images. A copy of the written consent is available for review by the Editor-in-Chief of this journal.

## Competing interests

The authors declare that they have no competing interests.

## Authors’ contributions

The author YWK was involved in drafting the manuscript or revising it. JHL and SLH obtained the data and figures, and drafted the manuscript and references. KSC made the study design and concept, drafted the manuscript, and made a critical review. All authors read and approved the final manuscript.
